# Preclinical Toxicity and Safety of MM-129—First-in-Class BTK/PD-L1 Inhibitor as a Potential Candidate against Colon Cancer

**DOI:** 10.3390/pharmaceutics13081222

**Published:** 2021-08-07

**Authors:** Justyna Magdalena Hermanowicz, Bartlomiej Kalaska, Krystyna Pawlak, Beata Sieklucka, Joanna Miklosz, Mariusz Mojzych, Dariusz Pawlak

**Affiliations:** 1 Department of Pharmacodynamics, Medical University of Bialystok, Mickiewicza 2C, 15-222 Bialystok, Poland; bartlomiej.kalaska@umb.edu.pl (B.K.); beata.sieklucka@umb.edu.pl (B.S.); joanna.miklosz@umb.edu.pl (J.M.); dariusz.pawlak@umb.edu.pl (D.P.); 2 Department of Clinical Pharmacy, Medical University of Bialystok, Mickiewicza 2C, 15-222 Bialystok, Poland; 3 Department of Monitored Pharmacotherapy, Medical University of Bialystok, Mickiewicza 2C, 15-222 Bialystok, Poland; krystyna.pawlak@umb.edu.pl; 4 Department of Chemistry, Siedlce University of Natural Sciences and Humanities, 3 Maja 54, 08-110 Siedlce, Poland; mariusz.mojzych@uph.edu.pl

**Keywords:** anticancer drug, zebrafish, safety profile, colon cancer, pharmacokinetic

## Abstract

MM-129 is a novel inhibitor targeting BTK/PI3K/AKT/mTOR and PD-L1, as it possesses antitumor activity against colon cancer. To evaluate the safety profile of MM-129, we conducted a toxicity study using the zebrafish and rodent model. MM-129 was also assessed for pharmacokinetics features through an in vivo study on Wistar rats. The results revealed that MM-129 exhibited favorable pharmacokinetics with quick absorption and 68.6% of bioavailability after intraperitoneal administration. No serious adverse events were reported for the use of MM-129, confirming a favorable safety profile for this compound. It was not fatal and toxic to mice at an anticancer effective dose of 10 μmol/kg. At the end of 14 days of administering hematological and biochemical parameters, liver and renal functions were all at normal levels. No sublethal effects were either detected in zebrafish embryos treated with a concentration of 10 μM. MM-129 has the potential as a safe and well-tolerated anticancer formulation for future treatment of patients with colon cancer.

## 1. Introduction

In the drug development process, it is crucial to anticipate possible side effects and predict toxicity before starting clinical trials of potential drug candidates. Drug toxicity is a key reason for drug failure in terms of clinical trials and drug withdrawal from the market. Modern pharmacotherapy based on extensive testing and validation of efficacy and safety utilizing various methodologies can increase the chances that a drug will reach consumers. 

1,2,4-triazine derivatives have been reported to possess a diverse spectrum of biological activities, including antifungal, antibacterial, anti-inflammatory, antianxiety, hypotensive, diuretic and anti-glaucomatous [1,2,3,4]. They are also of great interest in the field of medicinal chemistry as novel anticancer therapeutic agents due to their peculiar reactivity and mechanisms of action with respect to hitherto used drugs. Some derivatives of the pyrazolo[4,3-e][1,2,4]triazine ring system were evaluated against different types of tumor cell lines including lung and breast colon and they showed antiproliferative activity already in the micromolar range [5,6,7]. MM-129 (pyrazolo[4,3-e]tetrazolo[1,5-b][1,2,4]triazine sulfonamide) is a chemical compound obtained by chemical synthesis. It has a similar chemical structure to seliciclib (roscovitine), which is the first selective oral cyclin-dependent kinase (CDK) inhibitor to enter the clinical trial (NCT03774446). Preclinical studies showed antitumor activity of seliciclib in a broad range of human tumor xenografts [8]. It plays a key role in regulating the cell cycle, promoting its progression or transition between the individual phases. It also blocks RNA II polymerase, lowers the expression of Bcl-2, Mcl-1, and XIAP genes, and increases p53 expression, which in turn leads to cell death via apoptosis. The results of the latest research indicate this compound, by inhibiting Cdk5 and blocking p53 degradation, significantly weakens programmed death-ligand 1 (PD-L1) expression, promoting anti-tumor immune response [9].

**Figure 1:**
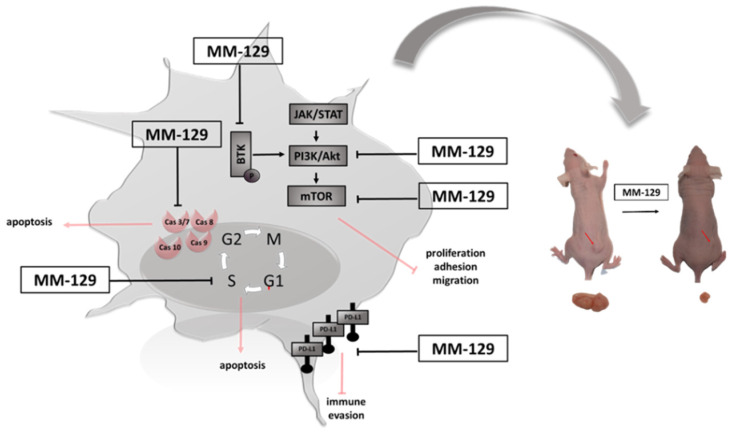
Schematic representation of possible anticancer mechanisms of MM-129. Abbreviations: BTK—Bruton’s tyrosine kinase; JAK—non-receptor tyrosine kinase; STAT—signal transducer and activator of transcription; mTOR—mammalian target of rapamycin; Akt—protein kinase B; PI3K—phosphoinositide 3-kinases; PDL1—Programmed death-ligand 1; MM-129—pyrazolo[4,3-e]tetrazolo[4,5-b][1,2,4]triazine sulfonamide.

We have recently shown that MM-129 effectively inhibits tumor development in both zebrafish xenografts and mice Foxn1nu/cmdb challenged with DLD-1 and HT-29 cells. We also found that it has the ability to reduction of PD-L1 expression with a simultaneous attenuate intracellular pathways promoting tumorigenesis inducing cell cycle arrest, like protein kinase B (Akt), mammalian target of rapamycin (mTOR), CDK2 and Bruton’s tyrosine kinase (BTK) (Figure 1) [10]. BTK is a member of the Tec family of nonreceptor protein tyrosine kinases which plays an important role in the development of B cell lymphoma and other solid tumors, including breast, ovarian, prostate and colon cancer [11,12,13]. Inhibition the activity of BTK through the use of ibrutynib or sorafenib effectively inhibits the neoplastic process. BTK inhibitors have been FDA-approved as the front-line treatment for B cell malignancy CLL/SLL. Future treatment strategy of solid tumors has yet to be fully evaluated [14,15].

Present mechanistic observation revealed that MM-129 possesses a cytotoxic, antiproliferative and proapoptotic activity and its effectiveness is much higher compared with the standard chemotherapy for colorectal cancer, i.e., 5-fluorouracil (5-FU) [16]. Despite promising pharmacological features, the possible toxic effects of MM-129 need to be carefully evaluated in order to optimize their design and applicability. The aim of the present study was to evaluate the pharmacokinetics features and safety profile of MM-129, a promising drug candidate against colon cancer. The toxicity study in zebrafish was also performed, which is acceptable and commonly used drug toxicity screening model. Short and long-term toxicities using BALB/ccmdb mice were conducted to determine the adverse effects of MM-129 when administered in a single dose, or in multiple doses during a period of 14 days respectively. In addition, we analyzed the effect of MM-129 on platelet functions and the coagulation system in rats.

## 2. Materials and Methods

### 2.1. Zebrafish Husbandry

The zebrafish embryos were maintained in an environmentally controlled room (28.0 ± 1.0 °C with a light/dark cycle) in accordance with the guidelines established by the Research Animals Department of the RSPCA (Royal Society for the Prevention of Cruelty to Animals). According to EU Directive 2010/63/EU, the earliest life-stages of zebrafish (embryo and eleutheroembryo cultures) are regarded as equivalent to an in vitro cell culture; therefore, they do not fall into the regulatory framework dealing with animal experiments. In our experiment we used zebrafish larvae younger than 120 hpf (hours post-fertilization); hence, an ethic approval was not required. Zebrafish embryos were obtained from mating adults, maintained and raised as described previously [17,18].

### 2.2. Zebrafish Toxicity Evaluation

**Figure 2:**
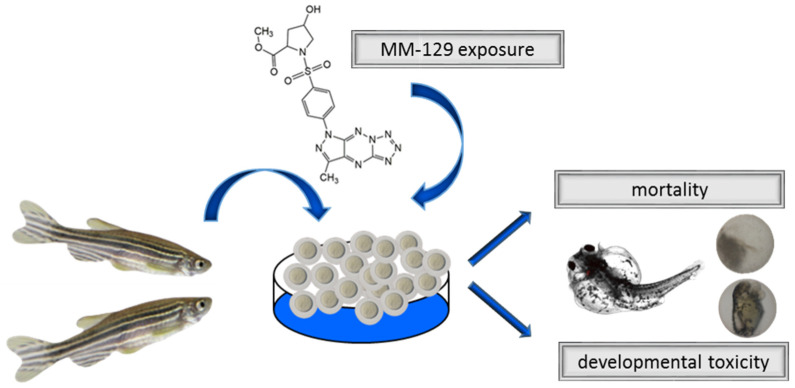
Schematic representation of exposure of zebrafish to MM-129. Abbreviations: MM-129—pyrazolo[4,3-e]tetrazolo[4,5-b][1,2,4]triazine sulfonamide.

The FET (Fish Embryo Toxicity) test was conducted with some modifications [19]. New fertilized wild type (WT) zebrafish embryos (0–2 hpf) exhibiting normal development or 72 hpf larvae were transferred to 24-well plates filled with a standard E3 medium and a series of concentrations of MM-129 (10, 30 and 100 μM, Department of Chemistry, Siedlce University of Natural Sciences and Humanities, Siedlce, Poland). Dimethyl sulfoxide (DMSO, Sigma-Aldrich, Saint Louis, MO, USA) was used as a drug solvent. The final concentration of DMSO in the wells did not exceed the damaging concentration of above 0.1%. The control embryos were incubated in an embryo medium in the presence of 0.1% DMSO. The embryos were inspected under stereomicroscope equipped with a camera at 24, 48, 72 and 96 h of treatment. The experiments were carried out in triplicate and 20 embryos were used for each group. Every 24 h, up to four apical observations were recorded as indicators of lethality: coagulation of fertilized eggs, lack of somite formation, lack of detachment of the tail-bud from yolk sac and lack of heart-beat. Early spontaneous movement rate after 24 h and hatching rate after 48, 72 and 96 h were also observed. Additional developmental alterations (heart rate, total body length) and embryo malformations, such as pericardial edema, yolk sac edema, tail curvature, somite formation and scoliosis, were recorded at 96 h (Figure 2).

For the toxicity test with 72 hpf larvae three replicates were performed. For each replicate, 20 objects were used in each concentration and 20 larvae were used as a control (0.1% DMSO). The larvae were monitored at 24 and 48 h after the treatment. The survival rate, heart rate, total body length and morphological deformities were examined and documented using a stereomicroscope equipped with a camera.

After completing the observations, all remaining embryos/larvae were euthanized using a buffered tricaine methanesulphonate solution as per the OECD test guideline 236 (Organization for Economic Co-operation and Development 2013).

### 2.3. Animals and Housing

A total of 12 male Wistar rats (pharmacokinetic study), 15 male Wistar rats (assessment of hemostatic parameters) and 64 Balb/ccmdb mice (toxicity study) were purchased from the Centre of Experimental Medicine at the Medical University of Bialystok and acclimated to the laboratory conditions for 3 days. The animals were housed in a standard 12 h light/dark cycle in temperature (22 ± 2 °C) and humidity (55 ± 5%) controlled room, were allowed free access to autoclaved standard pellet food and tap water throughout the experiments and grouped in cages as appropriate (Ssniff R-Z V1324). The animals’ health status was monitored throughout the experiments by a health surveillance program according to FELASA (Federation of European Laboratory Animal Science Associations) guidelines. The research protocol was approved by the Local Ethical Committee on Animal Testing (Permit No. 17/2019 (26.03.2019), 58/2020 (21.10.2020) and No. 5/WNP/2021 (17.02.2021) and conducted in accordance with ARRIVE (Animal Research: Reporting of In Vivo Experiments) guidelines, Directive 2010/63/EU of the European Parliament and of the Council on the protection of animals used for scientific purposes and the national laws.

### 2.4. Pharmacokinetics and Tissue Distribution of MM-129 in Rats

The rats were divided into three groups; the first group was given a single dose of 10 μmol/kg MM-129 intravenously via the femoral vein, the second group was given a single dose of 10 μmol/kg MM-129 intraperitoneally, and the third group was given a single dose of 10 μmol/kg MM-129 orally by gastric gavage. The dose was chosen based on our preliminary study, in which we assessed the anticancer activity of MM-129 [10]. MM-129 was prepared in a 10% DMSO. The rats were anesthetized by an intraperitoneal injection of pentobarbital (45 mg/kg), and blood was collected on K2-EDTA as anticoagulant via common carotid artery at 10, 20, 30, 60, and 120 min following MM-129 administration. After centrifugation at 10,000 rpm for 5 min at 4 °C, the plasma was transferred into polyethylene tubes and kept frozen until analysis at −80 °C. The rats were used to determine the tissue distribution of MM-129. The studied compound was administered in a single dose of 10 μmol/kg intraperitoneally. MM-129 was prepared as described above. The rats were anesthetized by an intraperitoneal injection of pentobarbital (45 mg/kg), and lungs, liver, spleen, brain, kidney, and small and large intestine were isolated rapidly from rats at 60 min following MM-129 administration, and immediately frozen until analysis at −80 °C.

The MM-129 concentrations were determined by high-performance liquid chromatography (HPLC). The chromatographic equipment included the Agilent Technologies 1260 series LC system composed of G1321 binary pump VL, G1379B degasser, G1329A autosampler, G1330B thermostat for autosampler, G1316A column thermostat and G1315C a diode array detector. The column effluent was monitored with diode array detector at 278 nm. The mobile phase was composed of 1.4% formic acid containing 30% of acetonitrile and it was pumped at a flow rate of 0.3 mL/min. In order to estimate MM-129 concentrations, the 100 μL plasma samples were deproteinized with 20 μL of 2 M perchloric acid, the 100 mg tissue samples were prepared by adding 300 μL of 20% trichloroacetic acid and centrifuged at 12,000× *g* for 30 min at 4 °C. The supernatant fluid was passed through WATERS 0.22 μm filters, and 5 μL was injected into the HPLC system for analysis. The prepared samples were separated on the Symmetry Waters C18 column (150 mm × 2.1 mm, 3.5 μm). Plasma concentration of MM-129 was expressed in µmol/L, whereas its levels in tissue samples were presented in nmol/g.

### 2.5. Unbound MM-129 Concentration Analysis

The unbound MM-129 concentration analysis was conducted using the rapid equilibrium dialysis device (Thermo Scientific, Waltham, MA, USA) according to the manufacturer instructions. Sodium citrate-anticoagulated pooled plasma was obtained from healthy rats by centrifugation of whole blood at 3500× *g* for 20 min at 4 °C. Spiked MM-129 rat pooled plasma samples (0.5 mL) at the concentration of 50 μM were dialyzed against phosphate buffered saline (0.75 mL) at pH 7.4 for 4 h. Total and unbound MM-129 concentration was measured using the HPLC method described above.

### 2.6. Toxicity Studies of MM-129 in Mice

**Figure 3:**
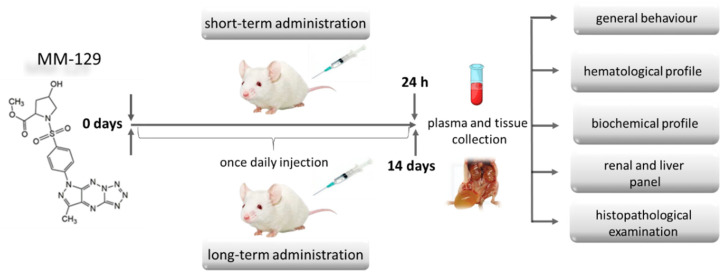
Schematic representation of the study protocol to evaluate the safety of MM-129 in short and long-term toxicity study in BALB/ccmdb mice. Abbreviations: MM-129—pyrazolo[4,3-e]tetrazolo[4,5-b][1,2,4]triazine sulfonamide.

Groups of 24 (short-term observation) and 40 (long-term observation) BALB/ccmdb (no significant differences between groups) aged 4–6 weeks were randomly divided into four groups (6 and 10 per experimental group respectively) and treated with a vehicle (control) alone (10% DMSO/phosphate-buffered saline (PBS), 0.2 mL) or MM-129 at a dose of 10, 20, 40 μmol/kg. These three doses were selected from the earlier toxicity study in the zebrafish model, where we have barely seen any sublethal alternations or mortality. More than 70% mortality rate observed for first 24 hpf in the highest concentration of 100 μM, prevented from using this concentration for further animal studies. In all groups, abnormal behavior, respiratory pattern, motor activities, reflexes, change in skin and fur, body weight changes, morbidity and mortality were monitored at 5, 15, 30, 45, 60 min and 24 h (24 h experiment) and once daily till the end of the experiment (14 days experiment) (Figure 3).

No sedation or anesthesia was used throughout the treatment period. Mice were electively anesthetized with a mixture of isoflurane and oxygen on day 2 or day 14 to assess the short and long-term toxicity respectively. A part of blood was taken from the right ventricle on the standard anticoagulant for the evaluation of hematological parameters: white blood cells (WBCs), red blood cells (RBCs), hemoglobin (HGB), hematocrit (HCT), mean corpuscular volume (MCV), mean corpuscular hemoglobin (MCH), mean corpuscular hemoglobin concentration (MCHC), platelets (PLT) in a blood analyzer (ABC Vet, Horiba, Germany). The remaining amount was centrifuged, and biochemical parameters: alanine aminotransferase (ALT), aspartate aminotransferase (AspAT), total bilirubin, creatinine (CREA), amylase (AMYL), blood urea nitrogen (BUN), lactate dehydrogenase (LDH), creatine kinase (CK) and inorganic phosphorus (Pi) were measured in serum by the automated clinical biochemical analyzer (Mindray BS 120, Darmstadt, Germany). At the time of necropsy, several tissues/multiple organs (kidney, spleen, liver, bone marrow) were collected from each mouse and evaluated for the presence of toxic lesions and collected immediately for histopathological examination. For histopathological studies, tissues were routinely placed in paraffin blocks and then sectioned by a Leica 2025 rotating microtome. Glass slides with affixed 4 μm-thick tissue sections were prepared, stained with (hematoxylin and eosin) H&E and evaluated under a light microscope.

### 2.7. Analysis of Hematological Parameters in Rats

A total of 15 male Wistar rats (272.2 ± 20.0 g) were anesthetized by intraperitoneal injection of pentobarbital (45 mg/kg). For the in vitro platelet aggregation study, the blood was collected into 3.13% trisodium citrate in a volume ratio of 9:1 from the heart of the rat. Platelet aggregation was measured after incubation of blood (500 μL) and MM-129 solution (500 μL) in the final concentration of 10 μM or 10% DMSO solution in 0.9% NaCl (500 μL) for 20 min at 25 °C, and then for 15 min at 37 °C. The changes in impedance were registered during 6 min after collagen addition (10 μg/mL) using Chrono-log aggregometer (Chrono-log Corp., Havertown, PA, USA).

For the in vivo study, the blood samples were collected from the hearts and the platelets were counted at 60 min following the intraperitoneal injection of the vehicle (10% DMSO/0.9% NaCl, 10 mL/kg) or MM-129 (40 μmol/kg, 10 mL/kg) in the blood analyzer (ABC Vet, Horiba, Germany). At the same time, platelet aggregation was measured after the incubation of the part blood (500 μL) and 0.9% NaCl solution (500 μL) according to the method described above. The remaining amount of sodium citrate anticoagulated blood was centrifuged at 3500× *g* for 10 min at 4 °C, and the plasma was deep-frozen (−80 °C) until further assays could be performed. Activated partial thromboplastin time (aPTT), prothrombin time (PT) and international normalized ratio (INR) was automatically determined by an optical method (Coag Chrom 4000, Bio-Ksel, Grudziadz, Poland), adding routine laboratory reagents (Bio-Ksel, Grudziadz, Poland).

### 2.8. Statistical Analysis

Shapiro–Wilk’s W test of normality was used for data distribution analysis. The normally distributed data were analyzed using a one-way analysis of variance (ANOVA) or unpaired Student *t*-test and shown as mean ± SD. Non-Gaussian data were presented as a median (full range) and analyzed using the non-parametric Kruskal–Wallis test or Mann–Whitney test. The Kaplan–Meier method was used for survival analysis. The statistical analysis was conducted using the GraphPad Prism software (Version 7.04). The differences were deemed statistically significant when *p* < 0.05.

## 3. Results

### 3.1. The Effects of MM-129 on Zebrafish Embryo/Larvae Development

#### 3.1.1. Embryos of 0–2 hpf

**Figure 4:**
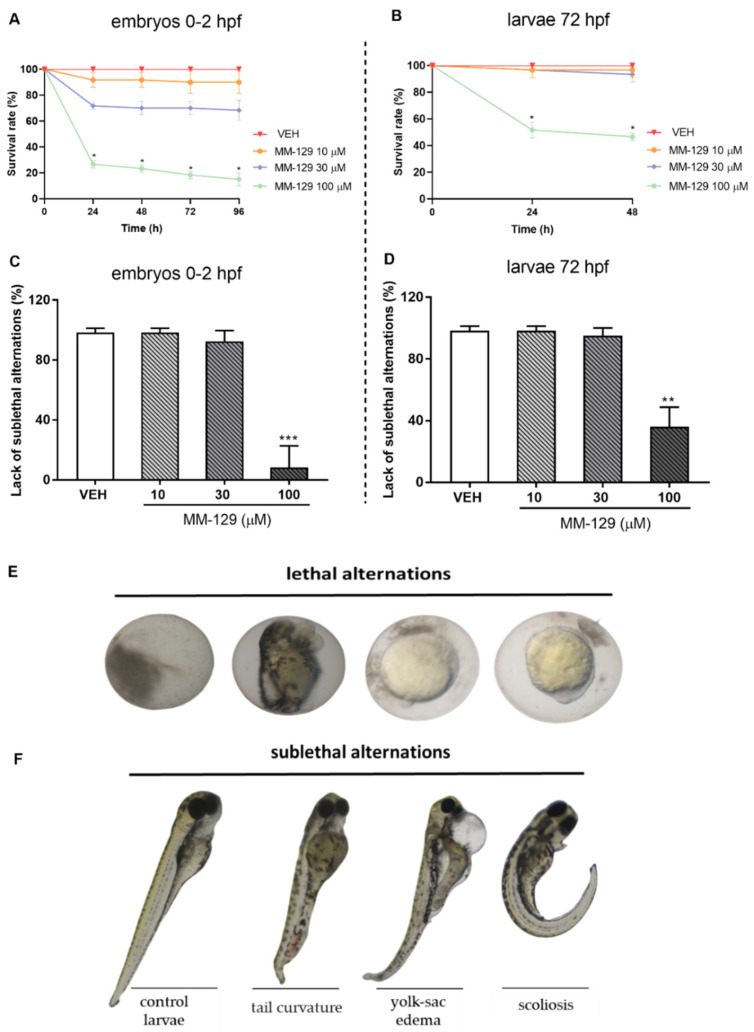
Survival rate (**A**,**B**) lack of sublethal alternations (**C**,**D**) for zebrafish embryos 0–2 hpf and larvae at 72 hpf exposed to MM-129 concentrations or vehicle (VEH) for 96 h. Lethal (**E**) and sublethal (**F**) alternations for zebrafish embryos 0-2 hpf exposed to MM-129 concentrations or vehicle (VEH) for 24 and 96 h. Sublethal alternations (**F**) for zebrafish embryos 0-2 hpf and larvae at 72 hpf exposed to MM-129 concentrations or vehicle (VEH) for 96 h. Data are shown as mean ± SD. * *p* < 0.05; ** *p* < 0.01; *** *p* < 0.001, vs. VEH within the group, *n* = 60 for each concentration. Abbreviations: MM-129—pyrazolo[4,3-e]tetrazolo[4,5-b][1,2,4]triazine sulfonamide; VEH—vehicle.

The survival and early embryonic development were examined at 24, 48, 72 and 96 h after exposure to MM-129. There were no significant differences between untreated embryos and embryos incubated with 0.1% DMSO. Mortality or developmental malformations in untreated embryos was not recorded at any time of the observation. Figure 4A show that at 96-h post-exposure the recorded survival was 90%, 68% and 15%, respectively in the group of embryos exposed to MM-129 at 10, 30 and 100 μM. The most prominent effects on the embryo survival and development were present at the beginning of treatment in these studied groups. Embryo phenotypic features were analyzed at 24 hpf (Figure 4E) and 96 hpf (Figure 4F). At the concentration of 100 μM malformations, such as spinal scoliosis, pericardial and yolk sac edema and tail curvature were observed (92%) (*** *p* < 0.001) (Figure 4C,F), whereas at the concentration of 30 μM and 10 μM these malformations were less frequent (8%) or none respectively.

**Figure 5:**
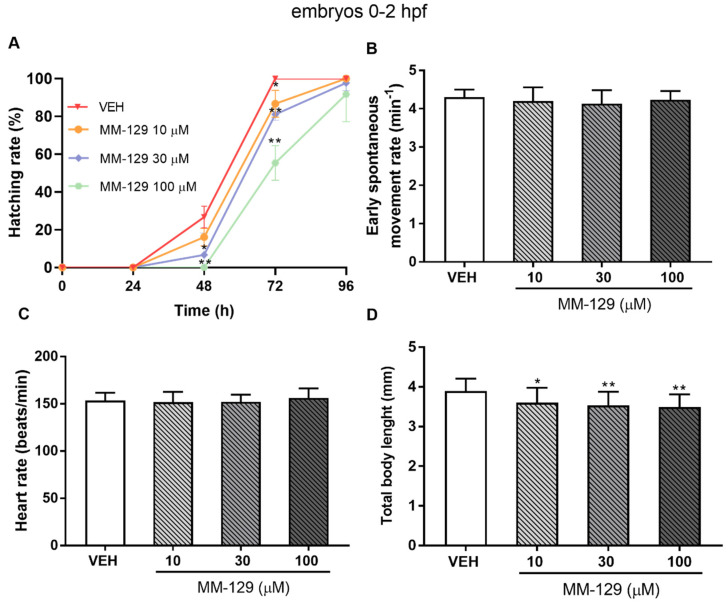
Hatching rate (**A**) for zebrafish embryos at 0–2 hpf exposed to MM-129 concentrations or vehicle (VEH) for 96 h; early spontaneous movement rate (**B**) for zebrafish embryos at 0–2 hpf exposed to MM-129 concentrations or VEH at 24 h of incubation; heart rate (**C**) and total body length (**D**) of zebrafish embryos 0-2 hpf at 96 h of incubation. Data are shown as mean ± SD; * *p* < 0.05; ** *p* < 0.01; vs. VEH within the group, *n* = 60 for each concentration. Abbreviations: MM-129—pyrazolo[4,3-e]tetrazolo[4,5-b][1,2,4]triazine sulfonamide; VEH—vehicle; hpf—hour post–fertilization.

The embryo hatching rate, determined by counting of zebrafish larvae outside the eggshell, was observed at 48 and 72 hpf, as hatching normally occurs during this period. The hatching rate of untreated embryos was approx. 27% at 48 hpf and 100% at 72 hpf. About 13% less of the embryos treated with 10 μM of MM-129, hatched at 72 hpf (* *p* < 0.05). MM-129 at the concentrations of 30 and 100 μM also delayed the hatching of 19% and 55% embryos respectively (** *p* < 0.01) (Figure 5A).

Spontaneous movement was recorded from 18 to 28 hpf. In control embryos, side-to-side alternating contractions initiated at 18 hpf, and gradually reached a peak of 4 bends/min at 24 hpf and then fluctuated in this way for nearly 4 h. In MM-129-treated embryos, a spontaneous movement initiated at a similar time and exhibited the same bending frequency in all studied groups (Figure 5B). Exposure to MM-129 did not affect the cardiac function of zebrafish embryos reflected as HR (heart rate) compared to the control at any time of the observations (Figure 5C). Interestingly, we noticed that embryo development was slowed when treated with 10 μM (* *p* < 0.05) and 30 μM (** *p* < 0.01) of MM-129, reflected as a reduction in total body length (96 hpf) (Figure 5D) and a higher proportion of chorionated embryos at 72 hpf (Figure 5A).

#### 3.1.2. Larvae of 72 hpf

Then, we performed a toxicity study for MM-129 in 72 hpf larvae, at the time when larvae were drug treated in most xenograft studies [16,20,21,22]. Increased mortality was observed only in the highest concentration of MM-129 (46%). In contrast, MM-129 added at concentrations of 10 and 30 μM did not cause significant larvae death throughout the assay (Figure 4B). About 64% of the larvae exposed to 100 μM of MM-129 showed body deformities (** *p* < 0.01). We observed body curvature as well as yolk sac edema. For the lower doses, no significant malformations or developmental abnormalities were observed during the evaluation until the end point (Figure 4D,F). MM-129 at all concentrations resulted no major changes at heart rate and total body length (Figure S1).

### 3.2. Pharmacokinetics of MM-129 in Rats

**Figure 6:**
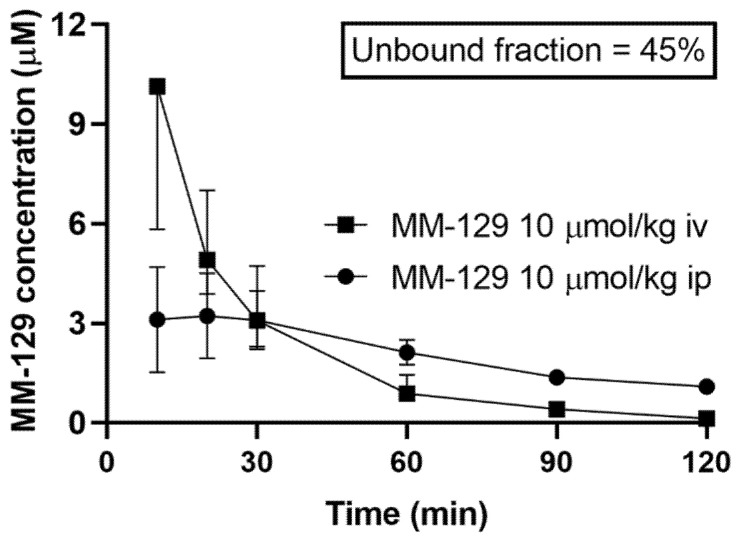
Pharmacokinetics curves of MM-129 in rats’ plasma. Results are presented as a median with lower and upper limits. Abbreviations: MM-129—pyrazolo[4,3-e]tetrazolo[4,5-b][1,2,4]triazine sulfonamide; iv, intravenous; ip; intraperitoneal.

MM-129 was administered to rats in the dose of 10 μmol/kg either intravenously, intraperitoneally, or orally to assess its pharmacokinetic properties. We found that MM-129 was quickly absorbed, with the time (Tmax) required to reach the maximum plasma drug concentration (Cmax 2.22–4.69 μmol/L) being 10–30 min after i.p. administration with relatively high bioavailability (68.6%). The plasma concentration-time curves of MM-129 after intravenous and intraperitoneal administration are shown in Figure 6.

The unbound fraction of MM-129 was 45%. It had an elimination half-life of 52–75 min after intraperitoneal administration (ip) at a dose of 10 μmol/kg. Area under the curve (AUC_0–120min_) ranged in value from 183 to 310 and 220 to 401 after intraperitoneal and intravenous administration respectively. The highest concentration measured at 60 min after a single dose of intraperitoneal administration of 10 μmol/kg MM-129 was observed in the small intestine, followed by the large intestine, kidney, lung, spleen, liver, and testicles. MM-129 levels in the brain were below the limit of detection. The pharmacokinetic parameters are listed in Table S1. The metabolism and elimination of MM-129 will be the subject of a separate study pending the identification of its key metabolites.

### 3.3. Toxicity Study of MM-129 in Mice

#### 3.3.1. Short-Term Administration

**Figure 7:**
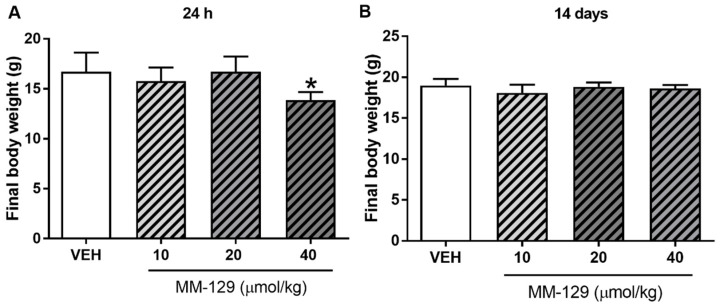
Final body weight of mice exposed to MM-129 concentrations or vehicle (VEH) after short-term administration (**A**) and long-term administration (**B**). Data are shown as mean ± SD; * *p* < 0.05 vs. VEH within the group *n* = 6–10 for each concentration. Abbreviations: MM-129—pyrazolo[4,3-e]tetrazolo[4,5-b][1,2,4]triazine sulfonamide; VEH—vehicle.

No deaths were reported during 24 h exposition. MM-129 at all doses did not produce signs of toxicity in mice over the observation period. Animals treated with higher doses showed transient sedation, ataxia, and inhibition of motor activity 15–30 min after administration. The effects were found to be dose-dependent, mild in the lower dose and marked in the higher dose. No clinical signs were observed in the skin and fur, eyes and mucus membrane (nasal), respiratory rate, autonomic effects (salivation, perspiration, piloerection, urinary incontinence, and defecation) and central nervous system (drowsiness, gait, ptosis, tremors and convulsion) among mice treated with MM-129. There were no significant MM-129-related effects on body weight in mice treated for 24 h at doses of 10, 20 μmol/kg. Only the highest dose resulted in slight/significant body weight loss (Figure 7A).

**Figure 8:**
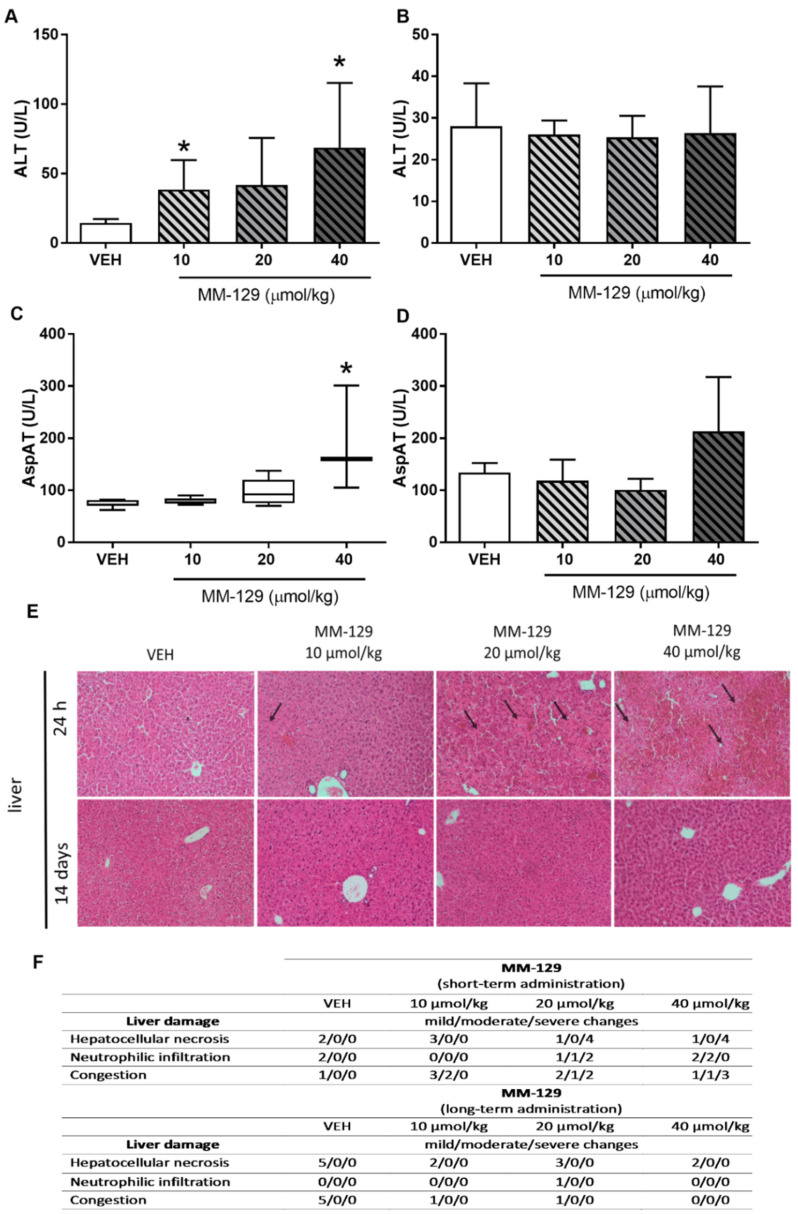
Alanine transaminase (ALT) (**A**,**B**) and aspartate transaminase (AST) (**C**,**D**) in mice treated with MM-129 concentrations or vehicle (VEH) after short-term administration (**A**,**C**) and long-term administration (**B**,**D**). Representative micrographs of tissue sections of liver, (original magnification, 10) (**E**) and selected clinical and gross necropsy observations of liver during safety study in mice after short-term and long-term administration of MM-129 and VEH (**F**). Original magnification, 10; hematoxylin and eosin staining. Data are shown as mean ± SD; * *p* < 0.05 vs. VEH within the group, *n* = 3–10. Abbreviations: ALT—alanine transaminase; AspAT—aspartate aminotransferase; MM-129—pyrazolo[4,3-e]tetrazolo[4,5-b][1,2,4]triazine sulfonamide; VEH—vehicle.

The hematological blood analysis did not reveal any important changes in all MM-129-treated groups. The morphological characteristics of animals undergoing the therapy were similar to untreated animals. Most of the parameters remained within the normal range in all animals. We observed only a slightly decreased level of MHCH after the administration of MM-129 at the doses of 40 μmol/kg (Table S2). Blood chemistry (BUN, CREA, AMYL, CK, Pi) did not change after single intraperitoneal administration of MM-129 at doses of 10, 20, and 40 μmol/kg (Table S3). We noted a significant/dose-dependent increase in ALT (alanine aminotransferase) after the administration of MM-129 at doses of 10 and 40 μmol/kg and an increase in AspAT (aspartate aminotransferase) after the exposition to the highest dose (Figure 8A,C). Additionally, this dose also resulted in a significant increase in total bilirubin (Table S3). These disturbances were confirmed in the histopathological examination of the liver (Figure 8E,F).

#### 3.3.2. Long-Term Administration

**Figure 9:**
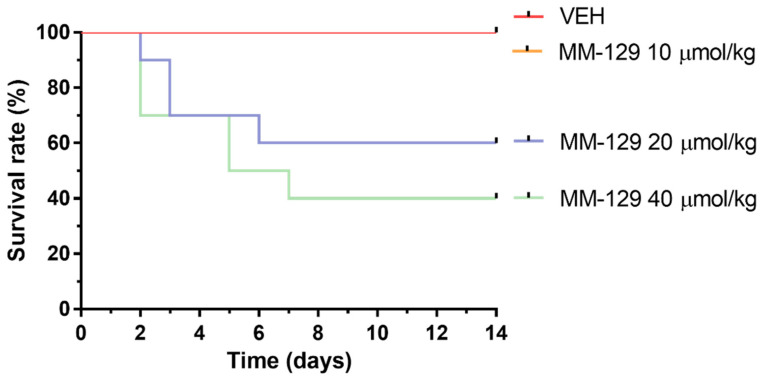
Survival rate of mice exposed to MM-129 concentrations and vehicle (VEH) after long-term administration. Abbreviations: MM-129—pyrazolo[4,3-e]tetrazolo[4,5-b][1,2,4]triazine sulfonamide; VEH—vehicle.

In the long-time study, MM-129 treatment-related mortalities were recorded in animals treated with a dose of 20 and 40 μmol/kg (Figure 9). The dose of 20 μmol/kg caused 40% of deaths in mice within the first week, and induced the incidence of low hypoactivity in some animals. At the higher dose of 40 μmol/kg, the mortality rate increased to 60% (6/10), and clinical signs of toxicity, such as hypoactivity, asthenia, and piloerection were more pronounced. MM-129 at a dose 10 μmol/kg did not produce mortality and signs of toxicity in mice over the observation period.

No significant differences were noted in mean baseline body weight between the randomized treatment groups. The mean body weight for control animals increased from 16.78 ± 0.97 g at a baseline to 18.63 ± 0.41 g at day 14. Virtually, all of the vehicle-treated as well as MM-129-treated mice gained weight during the observation period. Mean body weight gain was 1.7 ± 0.45 g and it was similar in mice receiving MM-129 at 10, 20 and 40 μmol/kg. There were no significant MM-129-associated effects on body weight in the surviving mice treated for 14 days at all doses (Figure 7B).

The values of hematological parameters of surviving animals treated with MM-129 were comparable with that of control (Table S2). MM-129 at a dose of 20 μmol/kg caused an increase in MCH and MCHC. Similarly, an increase in the value of MCHC was observed in animals treated with the highest dose. MM-129 did not induce treatment-related adverse effects with general behavior, and biochemical parameters (Table S3). No abnormalities were noticed in urinalysis (BUN and CREA) and in liver panel of treated animals compared with control animals (Figure 8B,D).

No erythroid, myeloid or lymphoid hypoplasia was encountered in the bone marrow of any of MM-129 treated mice (Table S4). There were proerythroblasts in all bone marrow smears. In most animals, a moderate number of megakaryocytes producing platelets and resting was present. In mice treated with a dose of 40 μmol/kg, an increased percentage of promyelocytes compared with the control group was found. However, this alternation was not reflected in peripheral blood parameters.

#### 3.3.3. Organ Morphology

**Figure 10:**
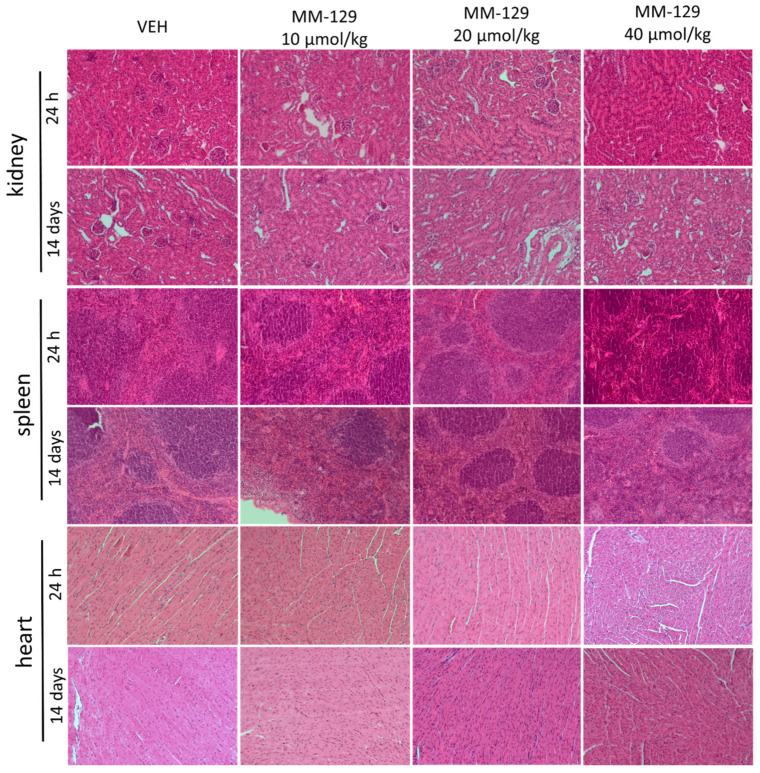
Representative micrographs of tissue sections of kidney, spleen and heart 24 h and 14 days after intraperitoneal administration of MM-129 at a dose of 10, 20, 40 μmol/kg and VEH in mice. Original magnification, 10; hematoxylin and eosin staining. Abbreviations: MM-129—pyrazolo[4,3-e]tetrazolo[4,5-b][1,2,4]triazine sulfonamide; VEH—vehicle.

Necropsy and histopathological examinations were performed on the liver, spleen, kidneys and heart of each animal to assess the damage to the internal organs or tissues after 24 h and 14 days after the termination of the experimental procedure. A routine histopathological examination showed normal spleen, kidneys and heart morphology of the vehicle-injected animals (Figure 10). The specimens of these organs in all MM-129 treated groups did also not show any pathological features, whereas MM-129 associated pathology of the liver tissue was seen in mice, after the first 24 h of the experiment. Though the image of the liver lobules construction was preserved, yet 24 h after MM-129 injection the analysis of liver showed a large area of hepatocyte necrosis, neutrophilic infiltration and hepatic hyperemia in animals treated with doses of 20 and 40 μmol/kg (Figure 8E,F). For both groups, severe necrotic changes of hepatocytes were found in 4/5 of the cases (80%). In turn, two and three animals showed mild necrotic changes, which were from the control and MM-129 (10 μmol/kg) treated groups respectively. In turn, all the submitted organs essentially showed normal histology after a long-term exposition to MM-129 at all doses.

### 3.4. The Effect of MM-129 on Platelet Function and Coagulation Parameters in Rats

MM-129 at a concentration of 10 μM did not change collagen-induced platelet aggregation in in vitro conditions. A similar effect was observed 1 h after the intraperitoneal administration of MM-129 at a dose of 40 μmol/kg. There was no statistical difference in PLT, aPTT, PT and INR in the rats treated with the tested drug (Table S5).

## 4. Discussion

The currently used therapies are often associated with major side effects and specific disorders, such as hand-foot syndrome or hematological disorders in the case of 5-FU or neuro-, oto- and nephrotoxicity in the case of oxaliplatin and with the appearance of the multidrug resistance (MDR) phenotype. The increase in the mortality rate, drug resistance phenomena and side effects of the current chemotherapeutic strategies against colorectal cancer (CRC) demand a necessity for intensified research with new drugs.

Our previous observations in in vivo conditions clearly showed that MM-129, a novel 1,2,4-triazine derivative at a dose of 10 μmol/kg, effectively inhibits tumor progression, leading to a significant reduction in tumor volume and mass in mouse xenografts [10]. Furthermore, it turned out to be more potent in inhibiting tumor growth compared with 5-FU [10,16].

Here, we aimed to estimate potential toxicity of MM-129 in the zebrafish model and its toxicokinetic profile in rodents. In recent years *Danio rerio* have been adopted as a model for the genetics study, drug development, toxicology, and drug safety screening [23]. It has become a prominent vertebrate model for assessing the toxicity of drugs and chemicals [24,25,26,27]. It possesses unique advantages: small size, ease of breeding, large numbers of progeny (high confidence in statistical analysis) and high grade of similarity [22]. Zebrafish genes have homologous to human genes of approximate 70%, they also possess similarities with respect to nervous and cardiovascular systems, liver, pancreas, intestine, gallbladder, and certain metabolic pathways [23]. Numerous studies indicate that drug toxicity screening with zebrafish is informative, and that this model can be used to predict some drug responses and toxicity in humans [28,29,30,31,32,33]. We examined the effects of various MM-129 concentrations on survival, morphological, and behavioral parameters in zebrafish embryos and larvae. Because previous observation of Gutiérrez-Lovera showed that the toxicity data of a compound evaluated in vitro and in a FET test at 0 hpf do not guarantee a reliable toxicity determination, and the additional toxicity studies using 72 hpf larvaes are necessary, we have selected two treatment time points for this model: 0–2 hpf stage (embryo) and 72 hpf stage (larvae) [21].

MM-129 at the highest used concentration (100 μM) demonstrated the lowest survival rate, regardless of zebrafish developmental stage and exposure time. However, the rates of lethality were more pronounced in embryo stage than in larvae. This is in line with the previous finding that early zebrafsh stages are more sensitive to external stimuli, including toxicants, chemicals, and mechanical stresses [34]. Additionally, MM-129 was previously reported to block cell division in the zebrafish embryo model at the 4-cell embryonic stage, which could have caused part of the embryos to slow development and death [16]. On the other hand, it is worth emphasizing that an anticancer effective dose of MM-129 (10 μM) did not affect the survival or embryonic development of zebrafish larvae, which indicates that it is non-toxic to normal cells retaining the ability to successfully inhibit tumor growth. In addition to the OECD test guideline 236 endpoints, we assessed developmental abnormalities which often preceded mortality to better understand the role that sublethal effects can have in the evaluation of toxicity from exposure to MM-129. The assessed sublethal endpoints included heart rate, pericardial and yolk sac edema, body curvature and length. Since the heart is the first internal organ to form and function during zebrafish development, at approximately 24–48 hpf, heartbeats can reflect toxicological impact [35]. Yolk sac edema is also an important toxicological endpoint because it provides a vital nutritive material for larval movement and plays an important role in the early development of zebrafish [36]. Pericardial and yolk sac edema, tail curvature, and spinal scoliosis only at the highest concentration of MM-129 were observed. Lower concentrations did not induce cardiac defects (typically manifested as pericardial edema) and other developmental abnormalities of zebrafish larvae at 96 hpf. MM-129 did not either affect the number of spontaneous movement, considered as a neurotoxicity marker in studies on behavioral changes due to chemical exposure [37]. Results from the zebrafish toxicity study clearly demonstrate a favorable safety profile of MM-129. Moreover, it seems to have better features in comparison with selicinib. Matrone et al. reported the absence of swimming activity, lack of heartbeat and tail blood flow at 57% of the embryos treated with selicinib at 5 μM [38]. It should be emphasized that this concentration is two times lower than the lowest of MM-129 used in our experiments. Furthermore, several larvae malformations, such as curved body (50%) and edema (82% for both mild and severe) was observed in the group of larvae exposed to selicinib at 5 μM. For comparison, MM-129 at concentrations 10 and even 30 μM resulted in no major changes in embryonic malformations or developmental abnormalities.

Animal models, despite some limitations, are still widely used in pharmaceutical research to predict human toxicity. Preclinical toxicology studies for novel anticancer agents are necessary to proceed to phase I clinical trials. The primary aim of safety evaluation is to identify a safe clinical starting dose and potential human toxicities. At the initial stage of rodent-based studies, we applied an analytical HPLC method to assess the pharmacokinetic properties of MM-129. It has a satisfactory preclinical pharmacokinetic profile, but after oral administration, its levels in plasma were below the limit of detection. The favorable pharmacokinetics of MM-129 after intraperitoneal administration provided a basis for choosing this route of administration in further experiments. We found that MM-129 is rapidly eliminated from the rat plasma with the circulation half-time of about 60 min. In turn, selicinib, which has a similar chemical structure to MM-129, is metabolized within the first 24 h. Moreover, the elimination half-life of CR8 the second-generation inhibitor of cyclin-dependent kinases derived from selicinib is longer and reaches about 3 h [39].

A new 1,2,4-triazine derivative at an antitumor effective dose of 10 μmol/kg was well tolerated up to 14 days of daily intraperitoneal injections. None of the animals showed signs of morbidity and there was no significant change in body weight gain. In the short-term and long-term safety study, no mortality at 10 μmol/kg was observed. In turn, higher doses of MM-129 (20 and 40 μmol/kg) caused an increase in animal mortality to 40% and 60% respectively with toxic clinical signs within the first days of long-term examination. Surviving animals treated with higher doses (but not 10 μmol/kg) showed a transient period of sedation, ataxia, and inhibition of motor activity for 15–30 min, which self-resolved. Importantly, these mice were then found to be normal in all respects, such as behavior and body weight until the end of the observation period. The results of gross and histopathology examination, blood cell counts, liver and renal functions were also all at normal levels.

MM-129 associated liver dysfunction was seen in mice, after the first 24 h of the experiment. After single administration, there was an increase in the biochemical (ALT, AspAT) and histopathological indicators of liver necrosis. The preexisting abnormal liver function led to the development of acute hepatotoxicity. These changes were likely the cause death of animals exposed to higher doses of MM-129. Hepatotoxicity related with chemotherapy regimens used in CRC is common, occurring in up to 47% of patients treated with 5-FU and 78% of patients treated with oxaliplatin-based chemotherapy [40]. Due to its anatomical location and unrepeatable function, the liver is exposed to a multitude of toxins and xenobiotics, including drugs. Cancer chemotherapy has evolved, giving newer medications that target cell biology a different pattern of liver toxicity. There are several hepatic conditions, such as sinusoidal obstructive syndrome, steatosis, and pseudocirrhosis which are more commonly associated with chemotherapy. These conditions can display clinical signs of acute hepatitis, liver cirrhosis, and even liver failure. Signs of liver disease are readily apparent by the detection of released hepatocellular transaminases ALT and AspAT into the serum, or by histologic examination of the biopsied liver tissue, which demonstrates a range of histologic changes, including steatosis, inflammation, ballooned hepatocytes, and fibrosis or cirrhosis [41]. Herein, disturbances in biochemical hepatic parameters were confirmed in histological examination of liver tissue, where dose-dependent necrotic hepatocytes death was detected. A high total bilirubin level in the group of animals treated with the highest dose of MM-129 was also noted. This observation is in line with Onishi et al., who reported that in addition to a typical hepatocellular injury, other presentations, including severe hyperbilirubinemia also commonly occur after chemotherapy [42]. It should be noted that no changes in biochemical parameters of liver function and any MM-129-related toxic liver lesions at the end of long-term exposition were found. These data suggest that MM-129 induced liver injury, especially at a dose of 10 μmol/kg was transient and fully reversible. A possible scenario for liver regeneration may result from functional compensation for the lost mass. Itoch et al. reported that upon acute or mild hepatocyte injury that affects only a limited population of hepatocytes, the remaining healthy hepatocytes proliferate and compensate the loss [43]. An interesting thing is that a recent clinical study with seliciclib also reported a reversible abnormal liver function in patients treated with doses of 800 mg twice daily [44]. This is also consistent with observations of others who reported that liver injury may be reversible, depending on the genetic variability, age, sex, and hepatic adaptation of the patient [45]. Furthermore, it was reported that patients with liver manifestations can be managed with supportive therapies, and liver toxicity may resolve after discontinuation of chemotherapy [46].

Contrary to 5-FU and seliciclib, MM-129-induced nephrotoxicity was not observed [8,47,48,49]. Renal panel assessed by BUN and CREA did not reveal impaired kidney function and hypokalemia. A histopathological examination of vital organs, including kidneys were conducted at the end of the treatment periods. The study showed normal architecture suggesting no morphological alterations of the spleen, heart and also kidney in relation to control groups. All these observations indicate a healthy status of kidneys in the MM-129 treated animals. Nephrotoxicity is common with the use of 5-fluorourcil and renal injury is its major drawback [47,50,51]. The available data indicate that oxidative stress plays a central role in mediating 5-FU-induced renal injury [52,53]. Seliciclib has also shown signs of toxicity, including hyponatremia, and hypokalemia, with other observed reactions, such as increased creatinine [49].

Hematological profiles of MM-129 treated rodents were similar to those of control animals. We did not observe any important changes in the biochemistry profile (excluding hepatic biochemical parameters) and in the blood cells count during the whole experiment in mice. We demonstrate that MM-129 at a therapeutic concentration and a maximum dose does not impair collagen-induced platelet aggregation, nor does it cause coagulation defects in rats. This is a favorable observation due to 5-FU-induced thrombotic complications and leukopenia, which remained a hallmark of treatment [54,55]. Leukopenia, leading to an immunocompromised state, can result in secondary pneumonia or sepsis [56]. Moreover, although treatment with seliciclib had no significant effect on peripheral blood cells obtained from a mouse model of glomerulonephritis, though a tendency toward a decrease in leukocyte counts was noticed [57]. Our preclinical toxicology studies within 14 days of treatment did not either reported anemia, leukocytosis or bone marrow hyperplasia. Myeloid cytopenias are the most common manifestations of chemotherapy-associated myelotoxicity. It is also one of the main reasons for dose modifications, dose delays, or discontinuation of therapy, potentially limiting the therapeutic benefit [58]. The absence of myelosuppression, observed in this study is clinically beneficial, especially because of negative impact of 5-FU—the first-choice chemotherapy drug for colon cancer on hematopoietic system has been reported [55,59,60]. Son et al. demonstrated that 5-fluorouracil caused bone marrow suppression severe leukopaenia and myelotoxicity in 7-week-old BALB/c mice after 5 days of exposure [61]. In turn, multiple administration of seliciclib at the dose of 350 mg/kg/day transiently decreased the BFU-E growth to less than 50% of the controls [62]. The toxicity of seliciclib to hematopoietic progenitors in vitro is within the same exposure range as cytotoxicity to cancer cells.

## 5. Conclusions

We found that MM-129 at an anticancer dose of 10 μmol/kg is not only effective but also very well tolerated. Lack of a negative effect of this dose on animal welfare was observed for the time period of 14 days. MM-129 displayed a favorable safety profile in both zebrafish and mouse toxicity screening models. It did not induce nephrotoxicity, changes in blood morphology, haemostatical and biochemistry parameters. MM-129-related disorders of hepatic function following acute exposure to a low dose were transient and fully reversible. Our observations suggest that MM-129 has a better safety profile than currently used drugs, including the reference drug 5-fluorouracil, and it seems to be a promising effective and safe candidate for further clinical development.

## Data Availability

Not applicable.

## Supplementary Materials

The following are available online at https://www.mdpi.com/article/10.3390/pharmaceutics13081222/s1, Figure S1: Heart rate (A) and total body lenght (B) of zebrafish larvaes 72 hpf at 96 h of incubation with MM-129. Data are shown as mean±SD; *n* = 60 for each concentration; Table S1: Pharmacokinetics and tissue distribution of MM-129 in rats; Table S2: Hematological analysis of whole blood samples from mice exposed to MM-129 concentrations or vehicle (VEH) after short-term and long-term administration; Table S3: Biochemical parameters in mice exposed to MM-129 concentrations or vehicle (VEH) after short-term and long-term administration; Table S4: Selected parameters of bone marrow examination of mice exposed to MM-129 concentrations or vehicle (VEH) after long-term administration; Table S5: The effect of MM-129 on platelet function and coagulation parameters in vitro and in vivo in rats.

## References

[1] Mullick P., Khan S.A., Begum T., Verma S., Kaushik D., Alam O. (2009). Synthesis of 1,2,4-triazine derivatives as potential anti-anxiety and anti-inflammatory agents. Acta Pol. Pharm..

[2] Kumar R., Sirohi T.S., Singh H., Yadav R., Roy R.K., Chaudhary A., Pandeya S.N. (2014). 1,2,4-triazine analogs as novel class of therapeutic agents. Mini-Rev. Med. Chem..

[3] Mamolo M.G., Falagiani V., Zampieri D., Vio L., Banfi E. (2000). Synthesis and antimycobacterial activity of some 4H-1,2,4-triazin-5-one derivatives. Farmaco.

[4] Cascioferro S., Parrino B., Spanò V., Carbone A., Montalbano A., Barraja P., Diana P., Cirrincione G. (2017). An overview on the recent developments of 1,2,4-triazine derivatives as anticancer compounds. Eur. J. Med. Chem..

[5] Mojzych M., Ceruso M., Bielawska A., Bielawski K., Fornal E., Supuran C.T. (2015). New pyrazolo[4,3-e][1,2,4]triazine sulfonamides as carbonic anhydrase inhibitors. Bioorg. Med. Chem..

[6] Mojzych M. (2011). Cytotoxic activity of some pyrazolo[4,3-e][1,2,4]triazines against human cancer cell lines. JCSP.

[7] Mojzych M., Tarasiuk P., Karczmarzyk Z., Juszczak M., Rzeski W., Fruzinski A., Wozny A. (2018). Synthesis, Structure and Antiproliferative Activity of New pyrazolo[4,3-e]triazolo[4,5-b][1,2,4]triazine Derivatives. Med. Chem..

[8] Benson C., White J., De Bono J., O’Donnell A., Raynaud F., Cruickshank C., McGrath H., Walton M., Workman P., Kaye S. (2007). A phase I trial of the selective oral cyclin-dependent kinase inhibitor seliciclib (CYC202; R-Roscovitine), administered twice daily for 7 days every 21 days. Br. J. Cancer..

[9] Dorand R.D., Nthale J., Myers J.T., Barkauskas D.S., Avril S., Chirieleison S.M., Pareek T.K., Abbott D.W., Stearns D.S., Letterio J.J. (2016). Cdk5 disruption attenuates tumor PD-L1 expression and promotes antitumor immunity. Science.

[10] Hermanowicz J.M., Pawlak K., Sieklucka B., Czarnomysy R., Kwiatkowska I., Kazberuk A., Surazynski A., Mojzych M., Pawlak D. (2021). MM-129 as a Novel Inhibitor Targeting PI3K/AKT/mTOR and PD-L1 in Colorectal Cancer. Cancers.

[11] Uckun F.M., Zheng Y., Cetkovic-Cvrlje M., Cetkovic-Cvrlje M., Vassilev A., Lisowski E., Waurzyniak B., Chen H., Carpenter R., Chen C.L. (2002). In vivo pharmacokinetic features, toxicity profile, and chemosensitizing activity of α-cyano-β-hydroxy-β-methyl-*N*-(2,5-dibromophenyl)propenamide (LFM-A13), a novel antileukemic agent targeting Bruton’s tyrosine kinase. Clin Cancer Res..

[12] Janda E., Palmieri C., Pisano A., Pontoriero M., Iaccino E., Falcone C., Fiume G., Gaspari M., Nevolo M., Di Salle E. (2011). Btk regulation in human and mouse B cells via protein kinase C phosphorylation of IBtkγ. Blood.

[13] Tankiewicz-Kwedlo A., Hermanowicz J.M., Domaniewski T., Pawlak K., Rusak M., Pryczynicz A., Surazynski A., Kaminski T., Kazberuk A., Pawlak D. (2018). Simultaneous use of erythropoietin and LFM-A13 as a new therapeutic approach for colorectal cancer. Br. J. Pharmacol..

[14] Akinleye A., Chen Y., Mukhi N., Song Y., Liu D. (2013). Ibrutinib and novel BTK inhibitors in clinical development. J. Hematol. Oncol..

[15] Messex J.K., Liou G.Y. (2021). Targeting BTK Signaling in the Microenvironment of Solid Tumors as a Feasible Cancer Therapy Option. Cancers.

[16] Hermanowicz J.M., Szymanowska A., Sieklucka B., Czarnomysy R., Pawlak K., Bielawska A., Bielawski K., Kalafut J., Przybyszewska A., Surazynski A. (2021). Exploration of novel heterofused 1,2,4-triazine derivative in colorectal cancer. J. Enzyme Inhib. Med. Chem..

[17] Westerfield M. (2000). The Zebrafish Book. A Guide for the Laboratory Use of Zebrafish (Danio rerio).

[18] Strähle U., Scholz S., Geisler R., Greiner P., Hollert H., Rastegar S., Schumacher A., Selderslaghs I., Weiss C., Witters H. (2012). Zebrafish embryos as an alternative to animal experiments—A commentary on the definition of the onset of protected life stages in animal welfare regulations. Reprod. Toxicol..

[19] Busquet F., Strecker R., Rawlings J.M., Belanger S.E., Braunbeck T., Carr G.J., Cenijn P., Fochtman P., Gourmelon A., Hübler N. (2014). OECD validation study to assess intra- and inter-laboratory reproducibility of the zebrafish embryo toxicity test for acute aquatic toxicity testing. Regul. Toxicol. Pharmacol..

[20] Rozkiewicz D., Hermanowic J.M., Tankiewicz-Kwedlo A., Siekluck B., Pawlak K., Czarnomysy R., Bielawski K., Surazynski A., Kalafut J., Przybyszewska A. (2020). The intensification of anticancer activity of LFM-A13 by erythropoietin as a possible option for inhibition of breast cancer. J. Enzyme Inhib. Med. Chem..

[21] Gutiérrez-Lovera C., Martínez-Val J., Cabezas-Sainz P., López R., Rubiolo J.A., Sánchez L. (2019). In vivo toxicity assays in zebrafish embryos: A pre-requisite for xenograft preclinical studies. Toxicol. Mech. Methods.

[22] Zhao C., Wang X., Zhao Y., Li Z., Lin S., Wei Y., Yang H. (2011). A novel xenograft model in zebrafish for high-resolution investigating dynamics of neovascularization in tumors. PLoS ONE.

[23] Zhang F., Qin W., Zhang J.P., Hu C.Q. (2015). Antibiotic toxicity and absorption in zebrafish using liquid chromatography-tandem mass spectrometry. PLoS ONE.

[24] Dooley K., Zon L.I. (2000). Zebrafish: A model system for the study of human disease. Curr. Opin. Genet. Dev..

[25] Cassar S., Adatto I., Freeman J.L., Gamse J.T., Iturria I., Lawrence C., Muriana A., Peterson R.T., Van Cruchten S., Zon L.I. (2020). Use of Zebrafish in Drug Discovery Toxicology. Chem. Res. Toxicol..

[26] Murugesu S., Khatib A., Ahmed Q.U., Ibrahim Z., Uzir B.F., Benchoula K., Yusoff N.I.N., Perumal V., Alajmi M.F., Salamah S. (2019). Toxicity study on Clinacanthus nutans leaf hexane fraction using *Danio rerio* embryos. Toxicol. Rep..

[27] Blomme E.A., Will Y. (2016). Toxicology Strategies for Drug Discovery: Present and Future. Chem. Res. Toxicol..

[28] Chakraborty C., Hsu C.H., Wen Z.H., Lin C.S., Agoramoorthy G. (2009). Zebrafish: A complete animal model for in vivo drug discovery and development. Curr. Drug Metab..

[29] Chen Y.H., Lee H.C., Hsu R.J., Chen T.Y., Huang Y.K., Lo H.C., Hu S.C., Harn H.J., Jeng J.R., Sun C.K. (2012). The toxic effect of Amiodarone on valve formation in the developing heart of zebrafish embryos. Reprod. Toxicol..

[30] Hermsen S.A., Pronk T.E., van den Brandhof E.J., van der Ven L.T., Piersma A.H. (2012). Concentration-response analysis of differential gene expression in the zebrafish embryotoxicity test following flusilazole exposure. Toxicol. Sci..

[31] Lee S.H., Kim H.R., Han R.X., Oqani R.K., Jin D.I. (2013). Cardiovascular risk assessment of atypical antipsychotic drugs in a zebrafish model. J. Appl. Toxicol..

[32] Shi X., Yeung L.W., Lam P.K., Wu R.S., Zhou B. (2009). Protein profiles in zebrafish (*Danio rerio*) embryos exposed to perfluorooctane sulfonate. Toxicol. Sci..

[33] Hung M.W., Zhang Z.J., Li S., Lei B., Yuan S., Cui G.Z., Man Hoi P., Chan K., Lee S.M. (2012). From omics to drug metabolism and high content screen of natural product in zebrafish: A new model for discovery of neuroactive compound. Evid.-Based Complement. Alternat. Med..

[34] Hill A.J., Teraoka H., Heideman W., Peterson R.E. (2005). Zebrafish as a model vertebrate for investigating chemical toxicity. Toxicol. Sci..

[35] Ismail H.F., Hashim Z., Soon W.T., Rahman N.S.A., Zainudin A.N., Majid F.A.A. (2017). Comparative study of herbal plants on the phenolic and flavonoid content, antioxidant activities and toxicity on cells and zebrafish embryo. J. Tradit. Complement. Med..

[36] De Oliveira G.A., de Lapuente J., Teixidó E., Porredón C., Borràs M., de Oliveira D.P. (2016). Textile dyes induce toxicity on zebrafish early life stages. Environ. Toxicol. Chem..

[37] Cheng R., Jia Y., Dai L., Liu C., Wang J., Li G., Yu L. (2017). Tris(1,3-dichloro-2-propyl) phosphate disrupts axonal growth, cholinergic system and motor behavior in early life zebrafish. Aquat. Toxicol..

[38] Matrone G., Mullins J.J., Tucker C.S., Denvir M.A. (2016). Effects of Cyclin Dependent Kinase 9 inhibition on zebrafish larvae. Cell Cycle.

[39] Sallam H., El-Serafi I., Meijer L., Hassan M. (2013). Pharmacokinetics and biodistribution of the cyclin-dependent kinase inhibitor -CR8- in mice. BMC Pharmacol. Toxicol..

[40] Alessandrino F., Qin L., Cruz G., Sahu S., Rosenthal M.H., Meyerhardt J.A., Shinagare A.B. (2019). 5-Fluorouracil induced liver toxicity in patients with colorectal cancer: Role of computed tomography texture analysis as a potential biomarker. Abdom. Radiol..

[41] Guicciardi M.E., Malhi H., Mott J.L., Gores G.J. (2013). Apoptosis and necrosis in the liver. Compr. Physiol..

[42] Onishi Y., Hatae M., Matsuda Y., Nakamura T., Kodama Y., Itoh M., Maruyama H., Maeda Y. (1995). Severe hyperbilirubinemia after cisplatin-based chemotherapy. Gan Kagaku Ryoho. Cancer Chemother..

[43] Itoh T., Miyajima A. (2014). Liver regeneration by stem/progenitor cells. Hepatology.

[44] Cicenas J., Kalyan K., Sorokinas A., Stankunas E., Levy J., Meskinyte I., Stankevicius V., Kaupinis A., Valius M. (2015). Roscovitine in cancer and other diseases. Ann. Transl. Med..

[45] Hamilton M., Wolf J.L., Rusk J., Beard S.E., Clark G.M., Witt K., Cagnoni P.J. (2006). Effects of smoking on the pharmacokinetics of erlotinib. Clin. Cancer Res..

[46] Sharma A., Houshyar R., Bhosale P., Choi J.I., Gulati R., Lall C. (2014). Chemotherapy induced liver abnormalities: An imaging perspective. Clin. Mol. Hepatol..

[47] Gelen V., Şengül E., Yıldırım S., Atila G. (2018). The protective effects of naringin against 5-fluorouracil-induced hepatotoxicity and nephrotoxicity in rats. Iran. J. Basic Med. Sci..

[48] Inoue K., Nagasawa Y., Yamamoto R., Omori H., Kimura T., Tomida K., Furumatsu Y., Imai E., Isaka Y., Rakugi H. (2009). Severe adverse effects of 5-fluorouracil in S-1 were lessened by haemodialysis due to elimination of the drug. NDT Plus.

[49] Zhelev N., Trifonov D., Wang S., Hassan M., El-Serafi I., Mitev V. (2013). From Roscovitine to CYC202 to Seliciclib—From bench to bedside: Discovery and development. Biodiscovery.

[50] Yousef H.N., Aboelwafa H.R. (2017). The potential protective role of taurine against 5-fluorouracil-induced nephrotoxicity in adult male rats. Exp. Toxicol. Pathol..

[51] El-Sayed E.-S.M., Abd-Ellah M.F., Attia S.M. (2008). Protective effect of captopril against cisplatin-induced nephrotoxicity in rats. Pak. J. Pharm. Sci..

[52] Rashid S., Ali N., Nafees S., Hasan S.K., Sultana S. (2014). Mitigation of 5-Fluorouracil induced renal toxicity by chrysin via targeting oxidative stress and apoptosis in wistar rats. Food Chem. Toxicol..

[53] Raghu Nadhanan R., Abimosleh S.M., Su Y.W., Scherer M.A., Howarth G.S., Xian C.J. (2012). Dietary emu oil supplementation suppresses 5-fluorouracil chemotherapy-induced inflammation, osteoclast formation, and bone loss. Am. J. Physiol. Endocrinol. Metab..

[54] Sougiannis A.T., VanderVeen B.N., Enos R.T., Velazquez K.T., Bader J.E., Carson M., Chatzistamou I., Walla M., Pena M.M., Kubinak J.L. (2019). Impact of 5 fluorouracil chemotherapy on gut inflammation, functional parameters, and gut microbiota. Brain Behav. Immun..

[55] VanderVeen B.N., Sougiannis A.T., Velazquez K.T., Carson J.A., Fan D., Murphy E.A. (2020). The Acute Effects of 5 Fluorouracil on Skeletal Muscle Resident and Infiltrating Immune Cells in Mice. Front Physiol..

[56] Kadoyama K., Miki I., Tamura T., Brown J.B., Sakaeda T., Okun Y. (2012). Adverse event profiles of 5-fluorouracil and capecitabine: Data mining of the public version of the FDA Adverse Event Reporting System, AERS, and reproducibility of clinical observations. Int. J. Med. Sci..

[57] Gherardi D., D’Agati V., Chu T.H., Barnett A., Gianella-Borradori A., Gelman I.H., Nelson P.J. (2004). Reversal of collapsing glomerulopathy in mice with the cyclin-dependent kinase inhibitor CYC202. J. Am. Soc. Nephrol..

[58] Kurtin S. (2012). Myeloid toxicity of cancer treatment. J. Adv. Pract. Oncol..

[59] Han Y., Yu Z., Wen S., Zhang B., Cao X., Wang X. (2012). Prognostic value of chemotherapy-induced neutropenia in early-stage breast cancer. Breast Cancer Res. Treat..

[60] Shitara K., Matsuo K., Takahari D., Yokota T., Inaba Y., Yamaura H., Sato Y., Najima M., Ura T., Muro K. (2009). Neutropaenia as a prognostic factor in metastatic colorectal cancer patients undergoing chemotherapy with first-line FOLFOX. Eur. J. Cancer.

[61] Son J.Y., Shin J.W., Wang J.H., Park H.J., Kim H.G., Raghavendran H.R., Son C.G. (2011). Chemotherapy-induced myelotoxicity and incidence of lung metastasis in an animal model. Hum. Exp. Toxicol..

[62] Song H., Vita M., Sallam H., Tehranchi R., Nilsson C., Sidén A., Hassan Z. (2007). Effect of the Cdk-inhibitor roscovitine on mouse hematopoietic progenitors in vivo and in vitro. Cancer Chemother. Pharmacol..

