# Supplementary material for: Preclinical Toxicity and Safety of MM-129—First-in-Class BTK/PD-L1 Inhibitor as a Potential Candidate against Colon Cancer
Source: Pharmaceutics. 2021 Aug 7;13(8):1222. doi: 10.3390/pharmaceutics13081222 (PMC8400941; doi:10.3390/pharmaceutics13081222)
Supplement: Supplementary file 1 [file pharmaceutics-13-01222-s001.zip › pharmaceutics-1312811-supplementary.pdf]

pharmaceutics

MDPI

Supplementary material: Preclinical Toxicity and Safety of MM-129—First-in-Class BTK/PD-L1 Inhibitor as a Potential Candidate against Colon Cancer

Justyna Magdalena Hermanowicz, Bartlomiej Kalaska, Krystyna Pawlak, Beata Sieklucka, Joanna Miklosz, Mariusz Mojzych and Dariusz Pawlak

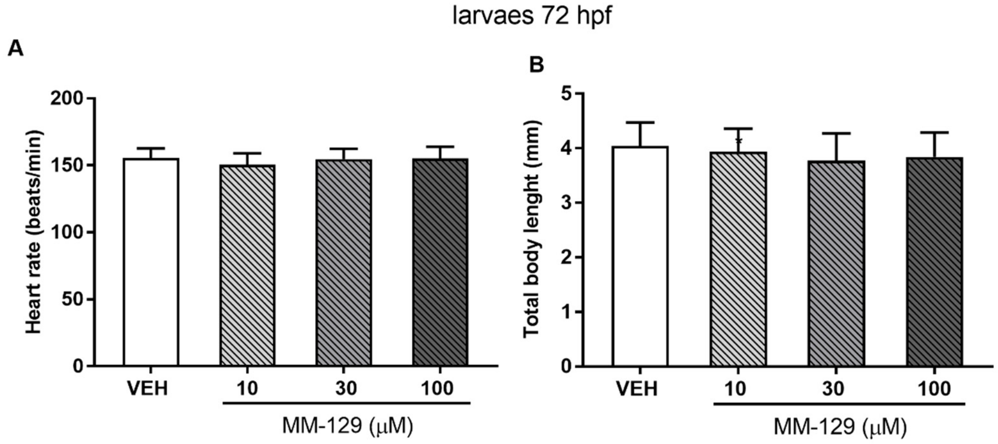

Figure S1. Heart rate (A) and total body lenght (B) of zebrafish larvaes 72 hpf at 96 h of incubation with MM-129. Data are shown as mean±SD; *n* = 60 for each concentration.
Abbreviations: MM-129 - pyrazolo[4,3-e]tetrazolo[4,5-b][1,2,4]triazine sulfonamide; VEH - vehicle; hpf - hour post–fertilization.

Table S1. Pharmacokinetics and tissue distribution of MM-129 in rats.

	**MM-129 10 µmol/kg iv**	**MM-129 10 µmol/kg ip**
CL (µmol/kg)/(µmol/L)/min	0.04 (0.03-0.05)	-
V_{ss} (µmol/kg)/(µmol/L)	0.60 (0.54-1.17)	-
Half life (min)	18.15 (12.74-25.84)	63.52 (51.54-75.49)
T_{max} (min)	10 (10-10)	20 (10-30)
C_{max} (µmol/L)	10.14 (5.83-10.28)	3.46 (2.22-4.69)
$AUC_{0\text{-}120min}$ (µmol/L*min)	360 (220-401)	247 (183-310)
$AUC_{0\text{-}\infty}$ (µmol/L*min)	360 (225-405)	346 (292-399)
Lung concentration (nmol/g of tissue)	-	0.46 (0.37-0.81)
Liver concentration (nmol/g of tissue)	-	0.17 (0.15-0.52)
Spleen concentration (nmol/g of tissue)	-	0.37 (0.33-0.46)
Brain concentration (nmol/g of tissue)	-	0 (0-0)
Kidney concentration (nmol/g of tissue)	-	0.81 (0.69-0.94)
Small intestine concentration (nmol/g of tissue)	-	2.78 (1.58-4.83)
Large intestine concentration (nmol/g of tissue)	-	1.17 (0.73-1.98)
Testicle concentration (nmol/g of tissue)	-	0.167 (0.06-0.60)

Data are shown as median with lower and upper limits.
Abbreviations: CL – clearance; Vss – steady state volume of distribution; T_{max} – time to reach maximum concentration; C_{max} – maximum concentration; AUC - area under the concentration–time curve; MM-129 - pyrazolo[4,3-e]tetrazolo[4,5-b][1,2,4]triazine sulfonamide

Table S2. Hematological analysis of whole blood samples from mice exposed to MM-129 concentrations or vehicle (VEH) after short-term and long-term administration.

		**short-term administration**		
	**VEH**	**MM-129 10 μmol/kg**	**MM-129 20 μmol/kg**	**MM-129 40 μmol/kg**
**WBC, 10^3/ mm³**	4.38±1.91	5.10±2.87	4.33±3.10	7.30±2.43
**RBC, 10^6/ mm³**	8.82±0.21	8.65±0.43	9.38±1.24	9.11±0.61
**HGB, g/dL**	14.4±0.38	13.7±0.70	15.0±2.07	714.5±0.99
**HCT, %**	49.5±1.10	48.3±2.24	53.7±7.63	51.5±3.59
**MCV, μm³**	56 (56-57)	55.5 (55-57)	57 (56-58)	56.5 (56-57)
**MCH, pg**	16.3±0.26	16.0±0.47	16.1±0.24	15.9±0.17
**MCHC, g/dl**	29 (28.5-29.8)	28.4 (27.8-28.8)	28 (27.8-29.3)	28 (27.8-28.2)*
**PLT, 10^3/mm³**	423 (350–496)	435 (312–502)	412.0 (351–627)	433 (360.0–628.0)
		**long-term administration**		
	**VEH**	**MM-129 10 μmol/kg**	**MM-129 20 μmol/kg**	**MM-129 40 μmol/kg**
**WBC, 10^3/ mm³**	4.99±2.68	7.07±3.36	8.00±1.46	9.33±0.97
**RBC, 10^6/ mm³**	8.98 (6.16-9.98)	8.71 (8.07-9.35)	8.77 (8.14-8.91)	8.23 (7.33-8.72)
**HGB, g/dL**	14.1±0.30	14.1±0.54	14.3±0.63	13.3±0.95
**HCT, %**	51.3 (35.5-56.9)	51 (46.3-53.5)	49.8 (46.5-52)	48.15 (41.9-49.9)
**MCV, μm³**	57 (55-59)	57 (56-59)	57 (57-60)	57.5 (57-60)
**MCH, pg**	15.6±0.48	16.0±0.28	16.6±0.32***	16.4±0.21**
**MCHC, g/dl**	27.4±0.50	28.0±0.65	28.7±0.55**	28.3±0.33
**PLT, 10^3/mm³**	466 (156–428)	421 (217–507)	426 (265–589)	481 (396–599)

Data are shown as medians with ranges or mean±SD; ** p<0.01; *** p<0.001 vs. VEH within the group, n =3-10.
Abbreviations: VEH – vehicle; WBC – white blood cells; RBC – red blood cells; PLT - platelets; HGB – hemoglobin; HCT - hematocrit; MCV – mean corpuscular volume; MCH – mean corpuscular hemoglobin; MCHC – mean corpuscular hemoglobin concentration; MM-129 - pyrazolo[4,3-e]tetrazolo[4,5-b][1,2,4]triazine sulfonamide.

Table S3. Biochemical parameters in mice exposed to MM-129 concentrations or vehicle (VEH) after short-term and long-term administration.

		**short-term administration**		
	**VEH**	**MM-129 10 μmol/kg**	**MM-129 20 μmol/kg**	**MM-129 40 μmol/kg**
ALT, U/I	14.40±2.97	38.40±21.3*	41.80±33.83	68.7±46.6*
AspAT, U/I	79.00 (62-82)	77 (72-90)	92 (70-137)	160.0* (105-301)
BUN, mg/dl	52.0±9.67	43.60±7.64	44.40±20.30	53.0±10.90
CREA, mg/dl	<0.46	<0.46	<0.46	<0.46
Total bilirubin, mg/dl	<0.15	<0.15	<0.15	1.93±1.18
AMYL, U/I	2141.0±132	1968.0±155	1615.0±36	1686.0±699
LDH, U/I	151.0±47.6	211.5±17.6	212.8±75.5	396.0±309
CK, U/I	92.60±7.57	109.0±27.7	120.0±31.1	132.0±48.2
Pi, mg/dl	8.95±0.5	8.50±0.62	7.82±0.63	8.60±0.28
		**long-term administration**		
	**VEH**	**MM-129 10 μmol/kg**	**MM-129 20 μmol/kg**	**MM-129 40 μmol/kg**
ALT, U/I	28.0±10.3	26.0±3.4	25.4±5.1	26.4±11.1
AspAT, U/I	134.0±18.4	118.0±40.5	101.0±21.3	212.0±105*
BUN, mg/dl	36.8±5.7	37.2±2.5	35.8±6.3	34.6±4.6
CREA, mg/dl	<0.46	<0.46	<0.46	<0.46
Total bilirubin, mg/dl	<0.15	<0.15	<0.15	<0.15
AMYL, U/I	1364.0±69.7	1429.0±231.0	1320.0±125.0	1325.0±232.0
LDH, U/I	312.0±115.0	249.0±63.1	290.1±38.5	522.0±179.0
CK, U/I	221.0±119.0	250.0±174.0	153.0±54.5	1082.0±1126
Pi, mg/dl	6.94±0.92	7.38±0.8	7.75±1.2	8.36±0.4

Data are shown as medians with ranges or mean±SD;. * p< 0.05 vs. VEH within the group, n =3-10.
Abbreviations: ALT – alanine transaminase; AMYL – amylase; AspAT – aspartate aminotransferase; BUN – blood urea nitrogen; CREA – creatinine; AMYL – amylase; LDH – lactate

dehydrogenase; CK – creatine kinase; Pi – phosphorus; MM-129 - pyrazolo[4,3-e]tetrazolo[4,5-b][1,2,4]triazine sulfonamide.

Table S4. Selected parameters of bone marrow examination of mice exposed to MM-129 concentrations or vehicle (VEH) after long-term administration.

**parameters (percentage)**	**VEH**	**MM-129 10 μmol/kg**	**MM-129 20 μmol/kg**	**MM-129 40 μmol/kg**
myeloblasts	0,552±0.5	0,47±0,16	1,06±0,515	0,742±0,517
promyelocytes	2,96±1.5	3,71±0.82	4,88±2.11	5,91±1.23*
myelocytes	10,7±1.33	10,9±2.2	13,8±2	13,3±5.13
metamyelocytes	14,1±1,94	14,3±1.7	13,6±1.09	13,1±1.34
neutrophils (bands)	13.8±3.1	15±2.64	13.8±1.65	13.9±3.47
neutrophils (segs)	12,9±3.8	12,2±1.93	13,1±2.49	11,6±3.63
proerythroblast	0,78±0.59	0,4±0.3	0,72±0.5	0,77±0.69
basophilic erythroblast	8,73±2.75	6,97±1.34	7,48±2.97	9,13±0.73
polychromatophilic erythroblast	10,3±1.47	9,97±1.02	8,54±2.7	9,86±2.88
acidophilic erythroblasts	12,1±2.26	12,5±1.48	11±1.53	9,19±1.74

Data are shown as mean ± SD; * $p < 0.05$ vs. VEH within the group, n =5.
Abbreviations: MM-129 - pyrazolo[4,3-e]tetrazolo[4,5-b][1,2,4]triazine sulfonamide; VEH – vehicle.

Table S5. The effect of MM-129 on platelet function and coagulation parameters *in vitro* and *in vivo* in rats.

	***in vitro* study**	
	**VEH**	**MM-129 10 μM**
	**Platelet aggregation**	
MaxA (Ω)	8.5 ± 1.3	8.3 ± 1.0
Slp (Ω/min)	2.9 ± 0.6	2.9 ± 0.6
Lag time (s)	90.9 ± 10.4	91.1 ± 7.4
AUC	24.9 ± 5.1	23.9 ± 3.5
	***in vivo* study**	
	**VEH**	**MM-129 40 μmol/kg**
Platelet count (10^3/mm^3)	511.0 (497.0-644.0)	573.0 (452.0-620.0)
	**Platelet aggregation**	
MaxA (Ω)	9.1 ± 1.5	8.7 ± 1.6
Slp (Ω/min)	3.3 ± 0.6	2.9 ± 0.6
Lag time (s)	103.2 ± 19.4	120.1 ± 30.0
AUC	25.1 ± 5.7	21.9 ± 6.4
	**Coagulation parameter**	
aPTT (s)	16.5 (14.0-20.9)	16.3 (13.8-24.0)
PT(s)	12.3 ± 0.4	12.0 ± 1.0
INR	1.4 ± 0.1	1.4 ± 0.1

The data were shown as median (full range) or mean ± SD, n=7.
Abbreviations: MaxA - maximal extension; Slp - slope of platelet aggregation; AUC - area under the curve; aPTT - activated partial thromboplastin time; PT - prothrombin time; INR - international normalized ratio.
